# The effect of Myo-inositol on sperm parameters and pregnancy rate in oligoasthenospermic men treated with IUI: A randomized clinical trial

**DOI:** 10.18502/ijrm.v17i10.5296

**Published:** 2019-11-07

**Authors:** Afsane Ghasemi, Fatemehsadat Amjadi, Seyedeh Masoumeh Ghazi Mirsaeed, Robabeh Mohammad Beigi, Samaneh Ghasemi, Yousef Moradi, Seyedeh Tahereh Ghazi Mirsaeed

**Affiliations:** ^1^Shahid Akbar Abadi Hospital Clinical Research Development Unit (ShACRDU), Iran University of Medical Sciences (IUMS), Tehran, Iran.; ^2^Cellular and Molecular Research Center, Iran University of Medical Sciences, Tehran, Iran.; ^3^Department of Anatomical Sciences, School of Medicine, Iran University of Medical Sciences, Tehran, Iran.; ^4^Department of Epidemiology, School of Public Health, Iran University of Medical Sciences, Tehran, Iran.

**Keywords:** Myo-inositol, Pregnancy, Sperm, Motility, Oligoasthenospermic.

## Abstract

**Background:**

In about 40% of the couples, the cause of infertility problems is attributed to men because of low sperm production and disturbed motility of sperm. Pieces of evidence show that Myo-inositol has a potential role for the treatment of sperm morphology and male fertility.

**Objective:**

This study aimed to determine the effect of Myo-inositol on the sperm parameters and fertility rate in patients with oligoasthenospermia treated by intrauterine insemination (IUI).

**Materials and Methods:**

This study was a randomized clinical trial conducted on 37 patients with oligoasthenospermia treated by IUI during 2016-2017. In this study, the patients were randomly divided into two groups of oligoasthenospermia treated with (Case group) and without Myo-inositol (Control group). The case group received 0.5 ml of Myo-inositol with a concentration of 2 mg/ml and incubated at 37°C incubator for 2 hr, but the control group had no interventions.

**Results:**

The results of this study showed that although there was no significant difference in sperm parameters including sperm motility and concentration before processing with Myo-inositol in the case group, but there was a significant increase in sperm motility during the treatment with Myo-inositol. The therapeutic effect of this method was confirmed on induction of pregnancy in 18% of the treated patients, in such a way that was about twice greater than those who did not receive the drug.

**Conclusion:**

According to the results of this study, the use of Myo-inositol is efficient enough to change sperm parameters to increase the chance of fertility.

## 1. Introduction

Myo-inositol is a sugar-like molecule considered as a member of the vitamin B complex. It is one of the most important cell membrane elements involved in the synthesis of lipids and cell growth and is also considered as one of the countless neurotransmitter precursors such as serotonin. Recently, various studies have shown that Myo-inositol is one of the precursors for the synthesis of phosphatidylinositol polyphosphates (1).

Myo-inositol plays a significant role in various processes associated with reproduction including gametes development, oocyte maturation, reproduction, and fetal development (2). Recent evidences indicate that Myo-inositol has a physiologic and therapeutic role in the improvement of human reproductive performance (3-5). Myo-inositol is also used to treat polycystic ovary syndrome (PCOS) and is taken into consideration as a method for induction of reproductive capacity. This substance is able to induce ovulation in ovaries and fertilization in patients with PCOS (6).

Considering the role of Myo-inositol in male fertility, it has been shown that Myo-inositol concentration in seminiferous tubules is higher from the serum (6). Thus, Myo-inositol concentration will increase by the movement of spermatozoa through epididymis and vas deferens (7).

The term "oligoasthenospermia" refers to the reduced number or movement ability of sperms and is a disorder that has been frequently reported in recent years. Accordingly, a change in sperm morphology can affect male fertility because of the production of Reactive Oxygen Species (ROS) affecting the motility and the morphology of sperms. Spermatozoa is covered by amorphous fibrosis in patients with oligoasthenospermia. It has also been shown that the treatment with Myo-inositol reduces the presence of amorphous fibrosis (8). On the other hand, Myo-inositol is able to regulate the osmotic property of the seminal fluid, while low osmolarity of this substance decreases the motility and the viscosity of sperm (9).

An increase in the amount of inositol monophosphates 1, an enzyme in dephosphorylating of phosphatidylinositol, is observed in patients with asthenozoospermia leading to the low motility of sperm (10, 11).

Evidence shows the potential role of treatment with Myo-inositol for improving the morphology of sperm and male fertility. Therefore, this study aimed to determine the effect of Myo-inositol on the sperm motility and the treatment of male infertility in order to indicate the role of this substance as a therapeutic method in male infertility.

## 2. Materials and Methods

This study was a double-blind randomized clinical trial conducted on 37 men with oligoasthenospermia treated with intrauterine insemination (IUI) during 2016-2017.

Our inclusion criteria were the couples with male factor infertility (oligoasthenospermia or asthenospermia) during the last 12 months, despite the normal female factors, hysterosalpingography, and hormonal tests. The exclusion criteria were severe oligo-astheno-teratospermia (OAT) or other male factors, untreated hormonal problems, BMI > 35, genital abnormalities, and partner women aged over 38 yr. The participants were initially evaluated through sperm analysis. In this regard, the term "oligospermia" refers to the sperm density of 5-20 million per milliliter, and "asthenospermia" refers to the sperm motility of < 40% with progressive motility (12).

**Figure 1:**
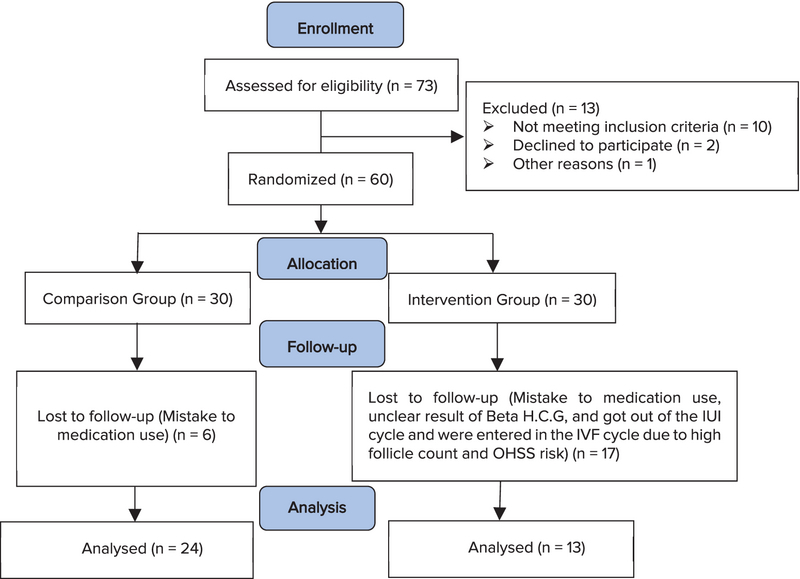
CONSORT diagram of the trial.

In the present study, 73 participants were enrolled initially, out of which 13 men were later excluded for falling under the exclusion criteria such as immigration to other cities and treatment discontinuation, not referring at the scheduled time, and not providing the results of their pregnancy test. Finally, 60 men remained to be randomly divided into the two groups (Figure 1).

### Preparation and analysis of sample semen

The semen sample for IUI treatment was collected through masturbation 48-72 hr after the last ejaculation and kept in a sterile container and placed at 37°C incubator for 30 min. Upon the liquefaction of the samples, sperm analysis was performed based on the version 2010 WHO criteria, and sperms were isolated from the plasma through the gradient method (sill-select plus commercial kit).

### Sperm wash through gradient method

At first, 1.5 ml of gradient medium with a concentration of 80% was poured into a falcon tube, and 1.5 ml of 40% gradient solution was slowly added into it, so that the 40% medium was completely at the top. Then, 2 ml of seminal fluid was slowly added to becentrifuged for 15 min at 3000 rpm. The sediment formed at the bottom of the tube was taken with a pipette and poured into another tube, and then 1.5 cm of sperm wash medium was added. It was also centrifuged at 3000 rpm for 5 min. After that, the top medium was taken with a pipette and 1.5 ml sperm wash medium was added again and centrifuged at 3000 rpm for 5 min. In the next stage, the upper medium was removed and the remaining sperms at the bottom of the tube were mixed with 1 ml of the medium and prepared for IUI.

### Intervention method

After the sperm separation, the participants were randomly divided into the two groups: the cases and the controls (n= 30/each) using a random number table. The control group had no intervention and the sperms were incubated for 2 hr at 37°C in 0.5 mL of the flushing medium in the special kit. In the case group, 0.5 ml of Myo-inositol at a concentration of 2 mg/ml was added to each men sperms, and they were incubated for 2 hr at 37°C incubator (the drug was licensed by the Ministry of Health with the IRC code of 7897458758743114 and the drug code of 104507 from Merck Company in Germany). After the incubation, the sperm parameters were re-examined and registered as triple blind. The studied parameters included the number of live sperms (viability), sperm motility, morphology, agglutination, volume, acidity, duration of liquefaction, DNA fragmentation index (DFI), and fertility.

### The sperm chromatin dispersion (SCD) test sperm chromatin dispersion test

To examine DNA fragmentation index (DFI) in both the groups, the SDFA kit (Sperm DNA Fragmentation Assay [Halosperm]) was used. In this test, sperm DNA and chromatin were denatured in a micro gel context affected by an acid treatment. In the next step, chromatin proteins were removed and DNA strands were spread around the sperm's head as far as possible, and they were visible as a halo around the sperm's head through coloration. Meanwhile, sperm DNA fragmentation led to the lack of the spread of DNA strands without observation of the halo, or with observation of very small haloes around the sperm's head. The normal range of this measurement is such a way that specimens with SDFA < 15% had a good rating and their DNA fragmentation rate was very low. But the SDFA from 15% to 30% had a low fragmentation rate (average rating) and SDFA > 30% had a high fragmentation rate (abnormal rating).

### Intrauterine insemination

The prepared specimens were inseminated into the uterus using a special IUI catheter. In this method, the sperms were washed in a sterile medium, and dead sperms and other cells were separated. The sperms prepared for insemination were passed through the cervix and injected directly into the uterus by a gynecologist using a catheter. This would shorten the pathway for the sperm to reach the ovum inside the uterine tube and would increase the probability of reaching the ovum. In addition, dead sperms and other cells as well as various materials that come with healthy sperms were separated, and thus, the active sperms were entered a nutritious environment that could easily move. Fifteen days later, serum B-HCG levels were checked for clinical pregnancy.

### Ethical consideration

This study was approved by the local ethics committee of Iran University of Medical Sciences (IR.IUMS.FMD.REC1396.9211290014) and registered in the Iranian Registry of Clinical Trials (IRCT20181031041519N1). In this study, informed consent was obtained from all participats in the study and their partners.

### Statistical analysis

The results were presented as mean and standard deviation (mean ± SD) for quantitative variables and as percentages for qualitative variables. Independent *t*-test and Chi-square test were used to compare quantitative and qualitative variables, respectively. Paired *t*-test and repeated measurement ANOVA was also used to determine the changes in the indices before and after the intervention. P < 0.05 was considered significant.

## 3. Results

**Figure 2:**
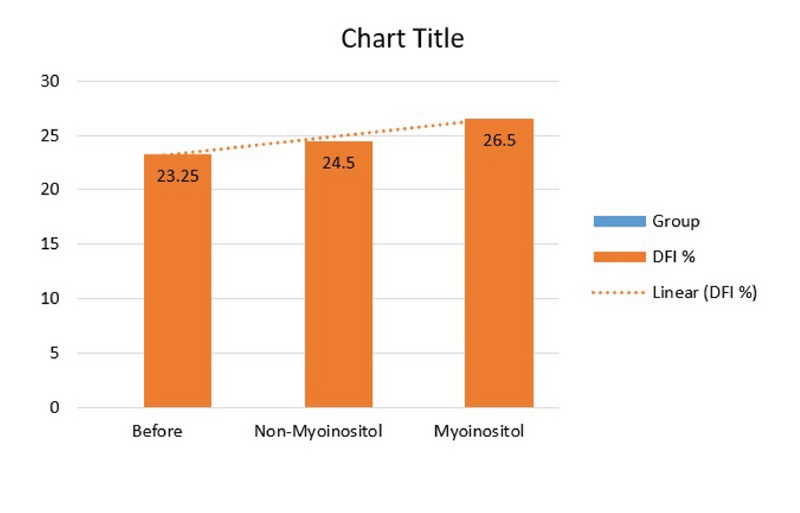
Sperm DFI examination in both the case and control groups (ANOVA test, F = 0.152, p = 0.014).

**Figure 3:**
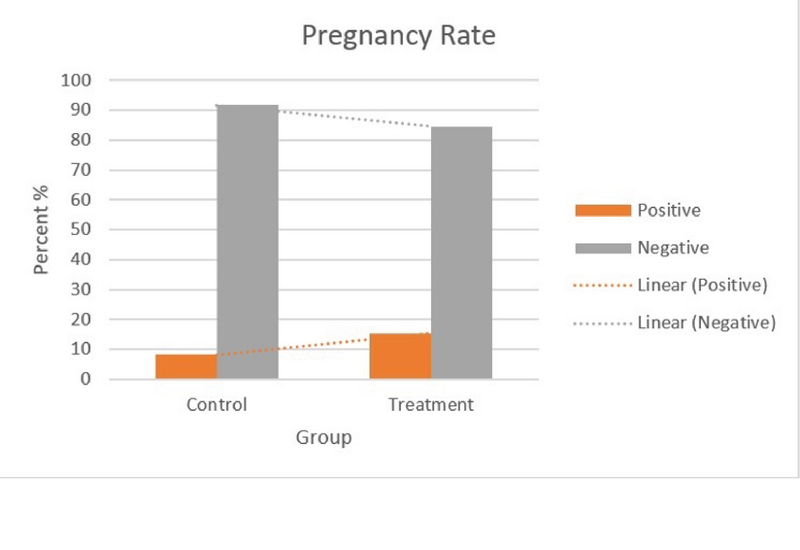
Pregnancy rate in the two groups (Independent *t*-test, df = 2, p = 0.050).

**Figure 4:**
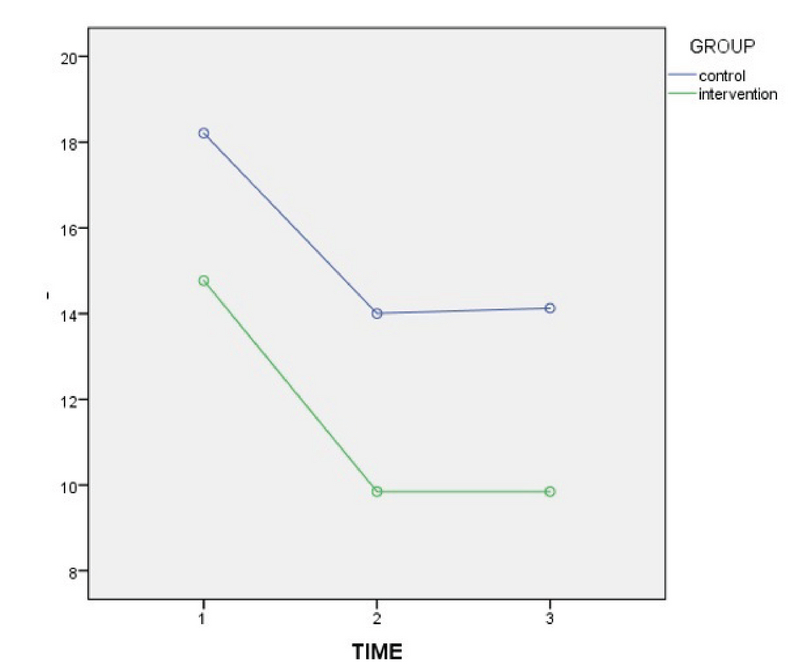
Comparison of the mean number of viability before and after treatment in the case and control groups.

**Figure 5:**
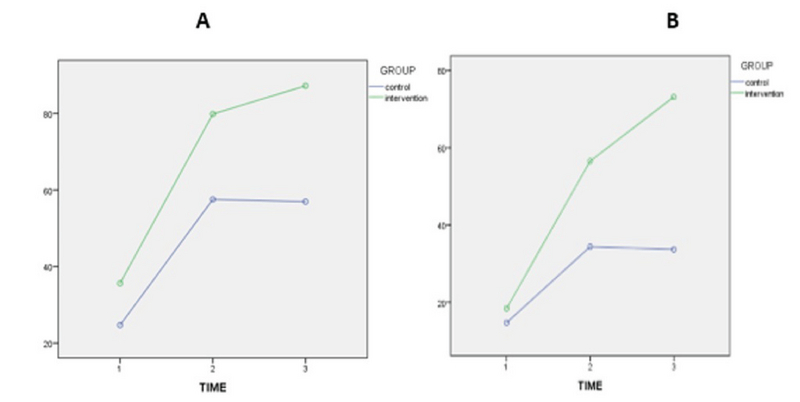
Comparison of the mean sperm motility (Figure A: Total mobility; Figure B: Progressive mobility) before, after, and 2 hr after the initial preparation stages in the case and control groups.

In the present study, 37 couples with male infertility (oligoasthenospermia), were enrolled into the two groups of with and without Myo-inositol treatment. Our results showed that the mean age of the women in the case and control groups were 29.92 ± 5.75 yr and 30.88 ± 3.54 yr, respectively, which was not significantly different. Also, the mean Body Mass Index (BMI) and infertility of the women were 23.92 ± 4.15 and 3.46 ± 2.71 in the case group and 22.92 ± 2.75 and 2.85 ± 2.64 in the control group, respectively (p = 0.362, p = 0.240) (Table I). In this study, the mean FSH, TSH, AMH, PRL, and follicles count of the women in the two groups were compared, and the results showed that these differences were not statistically significant in the two groups (Table I). On the other hand, Myo-inositol did not have any effect on the DFI increase and DNA damage (Figure 2). In this study, the mean sperm volumes in the case and control groups were 2.75 ± 1.07 ml and 2.58 ± 1.19 ml, respectively, without significant difference (p = 0.671). Also, the mean sperm liquefaction times in the control and case groups were 15.08 ± 3.54 min and 17.38 ± 9.87 min, respectively (p = 0.425). The acidity of sperm in the two groups showed no significant difference (p = 0.180) (Table II). The sizes of agglutination in both the case and control groups were also measured after the case. The results are shown in Table III without significant difference between the sizes of sperm agglutination in the two groups (p = 0.362). The treatment by Myo-inositol had a positive effect on 18% of the patients and increased the chance of fertility. This rate was lower in the control group (about 9%), which was statistically significant (p = 0.023) (Table III; Figure 3). The sperm concentration after sperm processing had no significant difference between the two groups before, after, and 2 hr after the initial preparation stages, and the treatment was not effective on the sperm concentration or sperm count (Figure 4; Table IV). Sperm motility was also examined and the results showed that there was a significant difference between the two groups, that is, the treatment increased sperm motility before, after, and 2 hr after the initial preparation stages (Figure 5; Table IV).

**Table 1:** Comparison of the baseline variables in the case and control groups


**Variables**	**Case group (n = 13)**	**Control group (n = 24)**	P-value* (95% CI)
**Age of women**	29.92 ± 5.75	30.88 ± 3.54	0.625 (-2.14, 4.04)
**Age of men**	36.08 ± 5.88	33.96 ± 4.68	0.624 (-5.70, 1.46)
**BMI**	23.92 ± 4.15	22.92 ± 2.75	0.240 (-3.30, 1.30)
**Infertility duration**	3.46 ± 2.71	2.85 ± 2.64	0.363 (-2.47, 1.25)
**Follicle**	3.07 ± 1.65	3.04 ± 2.13	0.959 (-1.42, 1.35)
**FSH**	6.80 ± 4.27	6.03 ± 2.27	0.694 (-4.91, 3.37)
**AMH**	6.21 ± 4.48	3.50 ± 3.09	0.272 (-7.92, 2.48)
**PRL**	11.57 ± 6.51	18.52 ± 7.65	0.243 (-6.90, 18.18)
**TSH**	1.59 ± 0.45	1.58 ± 0.34	0.954 (-0.43, 0.40)
Data presented as mean ± SD; *Independent *t*-test			
BMI: Body mass index; FSH: Follicle stimulating hormone; AMH: Anti mullerian hormone; PRL: Prolactin; TSH: Thyroid-stimulating hormone			

**Table 2:** Evaluation of sperm volume, PH, and liquefaction duration in the case and control groups


**Variables**	**Case group (n = 13)**	**Control group (n = 24)**	P-value* (95% CI)
**Vulome **	2.75 ± 1.07	2.58 ± 1.19	0.671 (-0.97, 0.63)
**Liquefaction duration**	15.08 ± 3.54	17.38 ± 9.87	0.425 (-3.48, 8.08)
**PH **	7.09 ± 0.09	7.038 ± 0.11	0.180 (-0.13, 0.02)
Data presented as mean ± SD; *Independent *t*-test			
PH: PH scale			

**Table 3:** Comparison of sperm agglutination in the case and control groups


**Variables**	**Case group (n = 13)**	**Control group (n = 24)**	P-value*
Agglotination			
Most	1 (7.7)	1 (4.2)	
Few	5 (38.5)	6 (25)	
Somewhat	2 (15.4)	1 (4.2)	
None	5 (38.5)	16 (66.7)	0.362
Results			
Positive	2 (15.4)	2 (8.3)		
Negative	11 (84.6)	22 (91.7)		0.023
Data presented as frequecy (%); *Chi-square test				

**Table 4:** Comparison of sperm parameters in the case and control groups


**Variables**	**Case group (n = 13)**	**Control group (n = 24)**	**P-value**
Motility			
Before	35.62 ± 9.73	24.71 ± 12.13	
After	79.85 ± 11.66	57.54 ± 25.74	
After 2 hr	87.23 ± 11.93	56.96 ± 26.67	0.0001
Sperm count (milion)			
Before	14.76 ± 2.29	18.20 ± 1.80		
After	9.84 ± 1.34	14.41 ± 1.51	
After 2 hr	9.84 ± 1.34	14.12 ± 1.39	0.453
Morphology (%)			
Before	0.77 ± 0.21	0.33 ± 0.09		
After	2.90 ± 0.42	1.77 ± 0.33	
After 2 hr	2.90 ± 0.42	1.77 ± 0.33	0.974

## 4. Discussion

The present study showed that the use of Myo-inositol was effective enough to change the properties of the sperm, leading to a high chance of fertility. Although, there was no significant difference in the sperm properties such as sperm motility before treating the sperm with inositol in the intervention group, there was a significant increase in the sperm motility after the treatment by Myo-inositol. Also, there was a significant improvement in the progressive sperm motility after treatment by Myo-inositol. This therapeutic method increased the fertility rate in the patients whose sperms were incubated with Myo-inositol by up to 18%, which was about twice greater than those who did not receive the drug. In the last few years, many scientific studies have been done and showed that Myo-inositol was one of the precursors for the synthesis of phosphatidylinositol (1). A growing number of studies about Myo-inositol support the theory of its physiological and therapeutic role in improving human fertility (3, 4). This finding has been widely studied in the field of increasing fertility through medical science and has been reported as a major regulator in a wide range of cellular processes such as gamete growth, oocyte maturation, fertility, and prenatal development (2). Myo-inositol has been specifically studied for the treatment of PCOS as a new method for ovulation induction. Myo-inositol restores spontaneous ovarian activity and increases the chances of fertility in patients with PCOS (9). Sperm dysfunction is one of the main causes of infertility, and its initial manifestation is low motility of sperms (13-15). Regarding the role of Myo-inositol in male fertility, Chauvin *et al*. showed that Myo-inositol concentration in seminal tubes was higher from the serum (16). In addition, it was reported that Myo-inositol increases the transmission of sperms through epididymis (7).

The sperms of patients with Oligoasthenospermia are completely covered with "fibers without amorphous materials" which reduces the cellular mobility. Clone *et al*. showed that the treatment with Myo-inositol could reduce the presence of these materials (8). It has also been observed that Myo-inositol plays an important role in the regulation of seminal fluid osmolarity since hypo- and hyper-osmotic conditions significantly reduce the motility and progressive speed of sperms (9). Myo-inositol increases the amount of inositol-1 Monophosphatase involving in the dephosphorylating of phosphatidyl inositol and plays a key role in the patients with Oligoasthenospermia (10). In fact, the change in the expression of IMPA-1 in the patients with Oligoasthenospermia can alter the signaling of phosphatidyl enzyme and leads to the low motility of sperm (10). Gulino *et al*. conducted another study in Italy and showed that the administration of Myo-inositol improved seminal fluid parameters in both the control and the oligoasthenospermia groups and was a useful treatment in increasing the probability of fertility in couples.

## 5. Conclusion

The present study showed a significant increase in their motility after treatment by Myo-inositol showing the efficacy of changes in the sperm properties induced by Myo-inositol leading to the high chance of fertility in couples. Furthermore, there was a significant improvement in the progressive sperm motility after treatment by Myo-inositol. The therapeutic effect of this method increased the fertility rate in patients whose sperms were incubated with Myo-inositol by up to 18%, which was about twice greater than those who did not receive Myo-inositol.

##  Conflict of Interest

The authors declare that they have no conflict of interest.
